# Supplementary material for: Recommendations of older adults on how to use the PROM ‘TOPICS-MDS’ in healthcare conversations: A Delphi study
Source: PLoS One. 2019 Nov 20;14(11):e0225344. doi: 10.1371/journal.pone.0225344 (PMC6867646; doi:10.1371/journal.pone.0225344)
Supplement: S1 File — (PDF) [file pone.0225344.s001.pdf]

Appendix file 1:

Round 1: online questionnaire

ROUND 1

Introduction

Many older persons have an appointment with a general practitioner, a specialist in a hospital or is a visited by a district nurse. At first your complaints will be discussed. But what else do you want to discuss?

The doctor or nurse (we call them 'healthcare professionals') will also ask you questions about other matters. For example, about your daily life or social situation. The healthcare professional will ask about these matters to get an idea about what is important to you, which can help to tailor treatments as best as possible to your personal situation.

But what should the healthcare professional exactly ask you about in order to get a good impression of your situation?

By completing this questionnaire, you help healthcare professionals (such as general practitioners, specialists and nurses) to ask the right questions.

By filling in the questionnaire, you give your permission for participation in the research. The data is processed anonymously and confidentially.

This research consists of three rounds:
Round 1: a questionnaire
Round 2: a group discussion with older persons and their family members
Round 3: a questionnaire

This is the first round. We will ask you at the end of the questionnaire if you want to participate in the second and third round. This is not mandatory.

This questionnaire consists of 23 questions and it takes about 10 minutes to compete this questionnaire.

We would like to thank you very much for your cooperation!

Personal information

1. Date of birth
   --/--/--

2. Gender
   - Male
   - Female

3. Postal code

4. In which country were you born?
   - Netherlands
   - Another country:

5. In which country was your father born?
   - Netherlands
   - Another country:

6. In which country was your mother born?
   - Netherlands
   - Another country:

7. What is the highest level of education that you have completed?
   - Fewer than 6 years of primary school
   - 6 years of primary school, lom school, mlk school (special education)
   - More than primary school / primary school without further completed education
   - Vocational school
   - Mulo / mms / mavo / secondary professional education
   - Hbs / gymnasium / atheneum (university entrance level)
   - University / tertiary education

8. What is your marital status?
   - Married
   - Divorced
   - Widow /widower / partner deceased
   - Unmarried
   - Long-term cohabitation, unmarried

9. What is your living situation?
   - Independent, alone
   - Independent, with others (partner, children, etc.)
   - Care home/residential care centre
   - Nursing home since

Overall wellbeing

10. Which report mark (1-10) would you give your overall wellbeing?

Where is this report mark based on? (For example: because I can do many things independently, etc.)

11. To what extend do you consider it to be important that the healthcare professional asks you about your functional limitations in daily life? You can think of walking, eating and getting dressed.

    - Not important
    - Slightly important
    - Neutral
    - Important
    - Very important

Why did you choose this answer?

12. To what extend do you consider it to be important that the healthcare professional asks you about your emotional wellbeing? You can think of nervousness, tranquillity and dreariness.

    - Not important
    - Slightly important
    - Neutral
    - Important
    - Very important

Why did you choose this answer?

13. To what extend do you consider it to be important that the healthcare professional asks you about your social functioning? You can think of visits to family members and/or friends.

    - Not important
    - Slightly important
    - Neutral
    - Important
    - Very important

Why did you choose this answer?

14. To what extend do you consider it to be important that the healthcare professional asks you about your quality of life?

- Not important
- Slightly important
- Neutral
- Important
- Very important

Why did you choose this answer?

15. To what extend do you consider it to be important that the healthcare professional asks you about your spirituality? You can think of zest of life, gratitude and the desire to achieve goals.

- Not important
- Slightly important
- Neutral
- Important
- Very important

Why did you choose this answer?

16. To what extend do you consider it to be important that the healthcare professional asks you about your environment? You can think of your living situation, safety, transport and support of friends and/or family.

- Not important
- Slightly important
- Neutral
- Important
- Very important

Why did you choose this answer?

17. Several topics were mentioned above, which can be discussed during a conversation with a healthcare professional. Would you like to indicate below which three topics are most important to you?

- Functioning in daily life
- Emotional wellbeing
- Social functioning
- Quality of life
- Spirituality
- Environment

18. Are there any other topics you would like to discuss with a healthcare professional?

Conclusion

19. Has someone helped you to complete this questionnaire?
    - No, I completed the list alone
    - Yes, somebody helped me to complete the list

20. If yes, what did the help consist of?
    - Someone else recorded the answers, but I selected the answers myself
    - I selected and recorded the answers together with someone else
    - Someone else selected and recorded the answers for me

21. Do you have any remarks/additions regarding the previously asked questions?

22. Between 29 May and 2 June, discussion group sessions will be organized with older persons and their families. The subject is: how to ask questions to older persons in a clear way. A discussion session will take one hour and a maximum of eight persons can participate. Simultaneously, the discussion session with (a maximum of eight) family members will be organized. The location will be chosen as centrally as possible depending on the residences of the participants. The travel costs will be reimbursed, and each participant receives a gift voucher of fifteen euros.

    Are you and your family member possibly be interested to participate in a discussion group session?

    Yes: Mail address: Residence: My family member will participate as well: Yes/No

23. On 19 June, the questionnaire will be sent for the third and final round of this research. May we also send you this questionnaire?
    NB. Everyone can participate in the third and final round, even if you do not want to participate in the discussion group session.

    Yes: Mail address
    No

Thank you very much for completing the questionnaire!

This research was set up and carried out by Vilans, a knowledge centre for long-term care, in collaboration with Radboud UMC, LUMC and Maastricht University. ZonMw finances this research. If you have any questions about this research, please contact Ruth Pel (r.pel@vilans.nl) or Cynthia Hofman (c.hofman@vilans.nl)
