# Supplementary material for: Recommendations of older adults on how to use the PROM ‘TOPICS-MDS’ in healthcare conversations: A Delphi study
Source: PLoS One. 2019 Nov 20;14(11):e0225344. doi: 10.1371/journal.pone.0225344 (PMC6867646; doi:10.1371/journal.pone.0225344)
Supplement: S2 File — (PDF) [file pone.0225344.s002.pdf]

Appendix file 2: Round 3: online questionnaire

ROUND 3

Introduction

In the first questionnaire we have asked you which topics you find important to discuss with a general practitioner or a nurse (we call them ‘healthcare professionals’) if you need care or a treatment.

We have discussed the results of all questionnaires with different groups of older persons. This provided explanations of what older persons consider as important topics. In this questionnaire we will ask you to read the explanations and indicate once again to what extend a topic is important to you.

In addition, certain questions from a questionnaire that are often left unanswered were discussed with older persons. Examples for not answering the questions are unclearly written questions and the use of difficult words. We have reformulated these questions and we would like to know whether you think that the reformulated questions are better than the original questions.

By completing in the questionnaire, you give your permission for participation in the research. The data is processed anonymously and confidentially.

We would like to thank you very much for your cooperation!

1. To what extend do you consider it to be important that the healthcare professional asks you about your functional limitations in daily life? You can think of walking, eating and getting dressed.

   An explanation of the older persons about this question:
   The participants indicated that this question makes clear whether help is needed. For example, the general practitioner can refer you towards other institutions for additional help. In addition, the participants mentioned that is sometimes difficult for the older persons to start talking about this topic and therefore it is nice when healthcare professionals ask about it.

   - Not important
   - Slightly important
   - Neutral
   - Important
   - Very important

The conversation about functional limitations in daily life includes the following questions:

Original question: Do you need help with combing your hair or shaving?
- Yes
- No

The participants noticed to following:
- Add the word 'with', most ladies don't shave

New question: Do you need help with combing your hair or with shaving?
- Yes
- No

Do you perceive the restated question to be clearer than the original question?
- Yes
- No

Explanation:

Original question: Do you use incontinence products?
- Yes
- No

The participants noticed the following:
- Give examples, for example, diapers, panty liners or Tenalady
- The question is too direct. We prefer: do you ever have unwanted urine loss when you laugh or cough?
- We feel embarrassed to answer this question

Restated question:

a. Do you have undesirable urine loss?
- Yes
- No
- Once in a while

b. Do you use something against the undesirable urine loss, for example, diapers, party liners or Tenalady?
- Yes
- No
- Not applicable

Do you perceive the restated question to be clearer than the original question?
- Yes
- No

Explanation:

Original question: Do you need help to get up out of a chair?
- Yes
- No

The participants noticed to following:
- Why getting up out of a chair? Can it also be a toilet, a bed or getting up from a couch?
- We would like to add the option ‘sometimes’, because for some older persons, it depends on how long they have been sitting if they need help to get up out of a chair

Restated question:
Do you need help with getting up from for example a chair of a couch?
- Yes
- No
- Sometimes

Do you perceive the restated question to be clearer than the original question?
- Yes
- No

Explanation:

Original question: Do you need help with taking your medicines?
- Yes
- No

The participants noticed to following:

- The word ‘taking’ can encompass many things, such as purchase medicine, prepare medicine, give medication by putting it in the mouth and make sure the patient swallows it.

Restated question: Do you need help with your medicines?
- Yes
- No

Do you perceive the restated question to be clearer than the original question?
- Yes
- No

Explanation:

---

2. To what extend do you consider it to be important that the healthcare professional asks you about your emotional wellbeing? You can think of nervousness, tranquillity and dreariness.

   An explanation of the older persons about this question:
   The participants indicated that they will not quickly raise this topic themselves. In addition, they find it very important that they have a good relationship with the healthcare provider. Lastly, the older persons think that the reason why they go to the healthcare professional is of importance. Healthcare professionals do not always have to ask about their emotional wellbeing.

   - Not important
   - Slightly important
   - Neutral
   - Important
   - Very important

The conversation about the emotional wellbeing includes the following questions:

Original question:
How often in the past months have you been very anxious?
- Always
- Very often
- Quite often
- Sometimes
- Almost never
- Never

The participants noticed to following:
- Rather “nervous” than anxious
- There are too many answer options

Restated question: How often have you been very nervous in the past month?

- Very often
- Sometimes
- Almost never

Do you perceive the restated question to be clearer than the original question?

- Yes
- No

Explanation:

Original question: How often in the past month have you felt sombre that nothing could cheer you up?

- Always
- Very often
- Quite often
- Sometimes
- Almost never
- Never

The participants noticed to following:

- We prefer other answer options, for examples with numbers
- Participants noticed that "nothing can cheer you up" is uncommon, there is always something that can cheer you up.

Restated question:
Mark with a cross on the ruler to indicate how often you felt sombre in the past month that almost nothing could cheer you up.

Never_________ Always

Do you perceive the restated question to be clearer than the original question?

- Yes
- No

Explanation:

---

3. To what extend do you consider it to be important that the healthcare professional asks you about your social functioning? You can think of visits to family members and/or friends.

   An explanation of the older persons about this question:

The participants brought forward that topics, such as loneliness can be discussed, and solutions can be provided. In addition, the participants feel more comfortable to talk about this topic with a healthcare professional they trust.

- Not important
- Slightly important
- Neutral
- Important
- Very important

---

4. To what extend do you consider it to be important that the healthcare professional asks you about your quality of life?

   An explanation of the older persons about this question:
   Participants mentioned that discussing quality of life only makes sense if the healthcare professional has enough time. By discussing quality of life, health professionals can look into whether older persons need help and who can possibly provide it.

   - Not important
   - Slightly important
   - Neutral
   - Important
   - Very important

The conversation about the quality of life includes the following questions:

Original question: Tick the box next to the sentence that best describes your health right now.
Pain/symptoms:

- I have no pain or other symptoms
- I have moderate pain or other symptoms
- I have very severe pain of other symptoms

The participants noticed to following:

- Two different things are asked for
- The participants find the wording ‘very severe’ doubled.

Restated question:
Tick the box next to the sentence that best describes your health right now.
Pain:

- I have no pain
- I have moderate pain
- I have severe pain

Do you perceive the restated question to be clearer than the original question?
- Yes
- No

Explanation:

Original question: Tick the box next to the sentence that best describes your health right now.
Mood:
- I am not anxious or despondent
- I am moderately anxious or despondent
- I am very anxious or despondent

The participants noticed to following:
- We would rather indicate how often this occurred. Therefore, we prefer the word ‘sometimes’ instead of the word ‘moderate’
- The question is too direct
- We would rather use the word ‘scared’ instead of the word ‘anxious’

Restated question:
Tick the box next to the sentence that best describes your health right now
Mood:
- I am not scared or despondent
- I am sometimes scared or despondent
- I am often scared or despondent

Do you perceive the restated question to be clearer than the original question?
- Yes
- No

Explanation:

Original question: Tick the box next to the sentence that best describes your health right now.
Brain functions such as memory, attention and thinking:
- I have no problems with my memory, attention and thinking
- I have some problems with my memory, attention and thinking
- I have severe problems with my memory, attention and thinking

The participants noticed to following:
- Participants find it difficult that three different things were asked within one question.
- Not everyone feels that forgetfulness is a problem. Therefore, it is important to not problematize the question.

- We would rather want to indicate how often we were bothered with something. Therefore, we would like to prefer the wording ‘sometimes’ instead of ‘some’.

Restated question: Tick the box next to the sentence that best describes your health right now.
Brain functions such as memory, attention and thinking:

How is your memory?
- Good
- Moderate
- Bad

How is your attention?
- Good
- Moderate
- Bad

How is your thinking?
- Good
- Moderate
- Bad

Do you perceive the restated question to be clearer than the original question?
- Yes
- No

Explanation:

Original question:
How is your quality of life in general?
- Excellent
- Very good
- Good
- Reasonable
- Poor

The participants noticed to following:
- We prefer to use ‘how do you perceive’ or ‘how satisfied are you’ instead of ‘how is’
- The participants indicated that quality of life is a vague concept. Providing examples would make it clearer.

Restated question: How do you perceive your quality of life? (For example, are you happy with your life as it is now, or how are you doing).
- Excellent
- Very good
- Good

- Reasonable
- Poor

Do you perceive the restated question to be clearer than the original question?
- Yes
- No

Explanation:

Original question:
Which report mark would you give your life at this moment?
Report mark: … Enter a figure between 0 and 10 here

The participants noticed to following:
- The participants preferred the wording ‘mark’ instead of ‘report mark’
- Using a yardstick would make it clearer

Restated question:
Which mark would you give your life at this moment?

0 ___________________10

Do you perceive the restated question to be clearer than the original question?
- Yes
- No

Explanation:

Original question:
How is your quality of life in general, in comparison to one year ago?
- Much better
- Slightly better
- About the same
- Slightly worse
- Much worse

The participants noticed to following:
- We would prefer to use the wording ‘how do you perceive’ instead of ‘how is’.
- It was not clear what was meant by one year ago. Do you mean a calendar year ago or 12 months ago?

Restated question:
How do you perceive your quality of life, in comparison to one year ago?
- Much better
- Slightly better

- About the same
- Slightly worse
- Much worse

Do you perceive the restated question to be clearer than the original question?

- Yes
- No

Explanation:

New topics came forward from the interviews and group discussion sessions with the older persons.

To what extend do you consider it to be important that the healthcare professional asks you whether you exercise frequently?

- Not important
- Slightly important
- Neutral
- Important
- Very important

To what extend do you consider it to be important that the healthcare professional asks you how you cope with stress?

- Not important
- Slightly important
- Neutral
- Important
- Very important

To what extend do you consider it to be important that the healthcare professional asks you how you deal with the health conditions and the effects on life?

- Not important
- Slightly important
- Neutral
- Important
- Very important

Conclusion

1. Has someone helped you to complete this questionnaire?
   - No, I completed the list alone
   - Yes, somebody helped me to complete the list

2. If yes, what did the help consist of?
   - Someone else recorded the answers, but I selected the answers myself
   - I selected and recorded the answers together with someone else
   - Someone else selected and recorded the answers for me

3. Do you have any remarks/additions regarding the previously asked questions?

Thank you very much for completing the questionnaire!

Please tick below:
- o I would like to receive my VVV gift voucher by post
- o I would like to receive my bol.com gift voucher by e-mail
- o I do not want a gift certificate

This research was set up and carried out by Vilans, a knowledge centre for long-term care, in collaboration with Radboud UMC, LUMC and Maastricht University. ZonMw finances this research.

If you have any questions about this research, please contact Ruth Pel (r.pel@vilans.nl) or Cynthia Hofman (c.hofman@vilans.nl)
