# Supplementary material for: Recommendations of older adults on how to use the PROM ‘TOPICS-MDS’ in healthcare conversations: A Delphi study
Source: PLoS One. 2019 Nov 20;14(11):e0225344. doi: 10.1371/journal.pone.0225344 (PMC6867646; doi:10.1371/journal.pone.0225344)
Supplement: Attachment — Submitted filename: 20191011 Response to reviewers.pdf [file pone.0225344.s004.pdf]

Academic Medical Center
University of Amsterdam

To the editor of PLOS ONE,

Amsterdam, 12 oktober 19

Dear Prof. Heber,

Thank you for reviewing our manuscript entitled '**Recommendations of older adults on how to use the PROM 'TOPICS-MDS' in healthcare conversations: a Delphi study'  PONE-D-19-16906**. We greatly appreciated the useful suggestions for improvement, and the opportunity to re-submit our manuscript. We have carefully studied all comments and suggestions, cumulating in a revised version of our manuscript. Enclosed you will find a point-by-point response.

All authors critically reviewed the manuscript and approved the final version.

We hope our manuscript now meets the criteria for publication in your journal.

On behalf of all authors,

Yours sincerely,

Ruth Pel-Littel, MSc

Correspondence to:
Ruth Pel-Littel
Academic Medical Centre Amsterdam
Department of Internal Medicine, section of Geriatric Medicine
PO Box 22660
1100 DD Amsterdam, The Netherlands

r.pel@vilans.nl

Ref.: Manuscript ID **PONE-D-19-16906**, entitled '**Recommendations of older adults on how to use the PROM 'TOPICS-MDS' in healthcare conversations: a Delphi study'**.

The authors' response can be found next to each comment. Corresponding revisions in the manuscript are addressed through track changes.

| **Reviewer comments** | **Authors' reflection** | **Revision** |
|---|---|---|
| Can I just check when I have done Delphi I have used quantative stats including Kendalls W and the McNemar test to verify items selected – I used a modified Delphi Okoli and Pawlowski, 2004 – but I wanted to check why you have not used any stats? | According to the article of Okoli and Pawloski, our Delphi was a 'ranking-type' Delphi since we sought group consensus about the relative importance of issues. We included the results of the Kendal's W, however, based on the low agreement scores the argument for using mainly descriptive statistics was confirmed. | Adjustments were made in line 41, 215 and 265:<br>**Consensus was pre-defined to be the point when >75% of the participants agreed that a domain was important or very important (scored on a 5-point Likert scale). The inter-expert agreement (Kendall's W) was for Round one 0.083 and for Round three 0.058. Based on these low inter agreement scores we further used descriptive statistics.** |
| Also Qualitative section page 10, I would like some more details for the reader.<br><br>The reader will need to know your paradigmatic position if you are presenting qualitative data so please add this. E.g., for the purpose of the qualitative analysis we have positioned ourselves as subtle-realists. This is important because it tells the reader what you present the information as you do. | To get a better understanding of the participants we described personal characteristics of the participants, such as age, education, cultural background. We did not focus explicitly on our paradigmatic position but focused on the purpose of collecting the qualitative data: to follow the Delphi process of response-analysis-feedback-response. However, for future studies it could be interesting to gather also information related to the paradigmatic position of all persons involved.<br><br>In our conclusion we added the recommendation addressing the diversity we found in the answers older persons gave (as | **216:**<br>The qualitative data comprised the in-depth explanations **of the motivations why participants ranked a domain as important to discuss in healthcare conversations. These data were collected with the purpose of enabling the participants of the following Delphi round to re-rank the domains with the group's viewpoint in mind.**<br><br>433:<br>**It would be interesting for future research to get a deeper insight in the differences** |

| | | |
|---|---|---|
| | shown in the low Kendal's W). For future research it could be interesting to gain a deeper insight in the differences between the specific groups (low/high educated, different cultural background) | **between these specific groups of older adults.** |
| For the themes can you consider if there is any sub-themes | This subsequently explains our choice to present the qualitative data according to the presented health domains, structured by the TOPICS-MDS. Because the Topics was our basis, we did not define subthemes. | **294:**<br>**Health domains to discuss in healthcare conversations: qualitative results**<br>Participants in Round 1 were invited to substantiate their assessment of health domains, both in the online questionnaire and in the focus groups. **The qualitative data are structured according to each health domain as described in the TOPICS-MDS: functional limitations, emotional wellbeing, social functioning and quality of life.** |
| Some readers will want to have a little more detail: e.g., when you mention limitations in daily life – what do you mean? I would expand the detail e.g., | We now introduced every health domain with a short explanation about the elements that form the domain so more detailed information is given. | **301: Functional limitations**<br>The domain of functional limitations includes basic and instrumental activities of daily life and mobility, such as walking(18).<br>**311: Emotional wellbeing**<br>The domain of emotional wellbeing concerns the mental state of a persons, e.g. feeling nervous, calm, happy, depressed etc.(18)<br>**325: Social functioning**<br>The domain of social functioning within the TOPICS-MDS inquires if a person feels impeded in entering into social contacts(18).<br>**337: Quality of life**<br>The domain of quality of life is composed of several attributes, such as mobility, self-care, usual activities, pain/discomfort, anxiety/depression and cognitive function(18). |

| | | |
|---|---|---|
| Think about a standard way to introduce the theme<br>identification of sub-themes focus on x most prevalent sub-themes<br>e.g., Functional limitations<br>This theme include X sub-themes: (a) the ability to walk to the shops – then include detail as above | See our reflection above. Unfortunately, we are not able to provide further detail on the numbers of how many participants mentioned sub-themes, since we did not inquire on this level of detail. However, this is a recommendation for future research. | |
| Also quotes can more detail so in analysis say how you identify individuals e.g., individuals will be identified by gender, age and educational level (school S, university U, higher degree;HD) an example of this would be (PM49U; meaning male participant, 49 years, University level education) | We provided in the section of the qualitative results more details about the participants now who provided the quotes, following the reviewers suggestions. | *Example:*<br>*307:*<br>*'I find it important to discuss functional limitations with my healthcare professional since he can arrange extra domestic help.'*<br>*(**female** participant**, 81 years** with a high level of education, online questionnaire).* |
| Reviewer #1: While some English editing would be useful, the current version is acceptable. I would suggest that the authors be advised of the contribution that an English editor might make to the quality of a manuscript. | The  last version (July 25, 2019)of this manuscript<br>was edited for proper English language, grammar, punctuation, spelling, and overall style<br>by one or more of the highly qualified native English speaking editors at AJE.<br>We attached the certificate of AJE to the submission files. We carefully checked again the manuscript for this resubmission. | |
