# Supplementary material for: Recommendations of older adults on how to use the PROM ‘TOPICS-MDS’ in healthcare conversations: A Delphi study
Source: PLoS One. 2019 Nov 20;14(11):e0225344. doi: 10.1371/journal.pone.0225344 (PMC6867646; doi:10.1371/journal.pone.0225344)
Supplement: Attachment — Submitted filename: 20190830 Response to reviewers.docx [file pone.0225344.s003.docx]

Academic Medical Center

University of Amsterdam

To the editor of PLOS ONE,

Amsterdam, August 30, 2019

Dear Prof. Heber,

Thank you for reviewing our manuscript entitled '**Recommendations of older adults on how to use the PROM ‘TOPICS-MDS’ in healthcare conversations: a Delphi study' PONE-D-19-16906**. We greatly appreciated the useful suggestions for improvement, and the opportunity to re-submit our manuscript. We have carefully studied all comments and suggestions, cumulating in a revised version of our manuscript. Enclosed you will find a point-by-point response.

All authors critically reviewed the manuscript and approved the final version.

We hope our manuscript now meets the criteria for publication in your journal.

On behalf of all authors,

Yours sincerely,

Ruth Pel-Littel, MSc

Correspondence to:

Ruth Pel-Littel
Academic Medical Centre Amsterdam
Department of Internal Medicine, section of Geriatric Medicine
PO Box 22660
1100 DD Amsterdam, The Netherlands
r.pel@vilans.nl

Ref.: Manuscript ID **PONE-D-19-16906**, entitled '**Recommendations of older adults on how to use the PROM ‘TOPICS-MDS’ in healthcare conversations: a Delphi study'**.

The authors’ response can be found next to each comment. Corresponding revisions in the manuscript are addressed through track changes.

| **Reviewer comments** | **Authors’ reflection** | **Revision** |
| --- | --- | --- |
| **Have the authors made all data underlying the findings in their manuscript fully available?  Reviewer #1: No** | The datasets used and/or analyzed during the study are available from the corresponding author on reasonable request. Optionally we also offer to upload the data on fig. share. | We added a statement on the availability of data and materials at the end of the manuscript:  *Availability of data and materials*  ***The datasets used and/or analyzed during the study are available from the corresponding author on reasonable request.*** |
| **Is the manuscript presented in an intelligible fashion and written in standard English?  Reviewer #1: No** | We sent the revised manuscript to the American Journal Experts for language editing | The manuscript has been revised on language by the American Journal Experts . |
| **- The tool is titled for older adults and informal caregivers survey - these are two distinct population groups. Was there a difference in the findings?** | The TOPICS-MDS exists of two surveys, one for older persons and one for informal caregivers (Lutomski et al., 2013). For older persons data was collected on demographics, morbidity, quality of life, functional limitations, emotional wellbeing, social functioning and health services utilization. For informal caregivers data was only collected on demographics, hours of informal care and quality of life.  For this study we have chosen to focus on the views of older persons themselves because we think in health care conversations the primary focus is on health related domains of the older patients addressed by the professionals. | We clarified this in the Introduction section and explained about the two different surveys within the TOPICS-MDS (line 79 - 81). In addition we specified this in the aim of the study (line 86):  ***The TOPICS-MDS comprises two*** ***multidimensional questionnaires: (1) one on the physical and mental health and wellbeing of older adults and (2) one on the caregiver burden and quality of life of informal caregivers.***  ***The aim of this study was to explore whether the TOPICS-MDS, the questionnaire for older adults, could facilitate SDM conversations between professionals and older adults with MCC.*** |
| **-If this tool is useful then the need to educate health care professionals is important and needs to be addressed. How might this be conducted?- there is no discussion of the implications of this study for the education of health care professionals specific to the use of this tool.** | We agree with the reviewer that educational aspects are important and that this can have more attention. We added the implications for practice in the discussion session. | We provided recommendations and examples regarding the education of health care professionals in how to use the TOPICS-MDS. In addition we describe how seniors' organisations put effort in the empowerment of older adults to prepare for a health care conversation (line 444- 456).  *Practice Implications*  ***The recommendations regarding the additional topics and the suggestions to improve the comprehensibility of the questions have been presented to the Dutch TOPICS Working Group. For the TOPICS-MDS to be used as input for individual healthcare conversations between older adults and healthcare professionals, the education of healthcare professionals is needed. Therefore, in the large scale SDM implementation program of the Dutch Care Institute (Zorg Instituut Nederland), a specific project has been designed to improve SDM with the PROM TOPICS-SF (Short Form), supported by the Dutch Geriatric Society. As a part of this project health professionals at geriatric hospital wards are supported in the use of the results of this PROM in individual health care conversations. For example, through e-learning, they learn how to use the PROM in healthcare conversations. In addition, the Dutch seniors' organisation KBO-PCOB and the Dutch migrant organization NOOM put a joint effort in the empowerment of older adults to prepare for a health care conversation with this PROM, with information sessions for older adults and educational material.*** |
| **- Was this a Delphi study, or was it simply three focus groups with specific intentions? I would suggest the former as it does appear that a consensus was sought. It does not appear from the reported findings that a group consensus was achieved, as different objectives were identified (lines 126-131).** | The design of the study was indeed a modified Delphi study, because we conducted several rounds with the same group of participants with the aim of gaining consensus. Within the rounds, different methods were used for some sub-groups of participants (low education, cultural diverse background), because we know that written internet surveys do not reach these subgroups and lead to low quality data. By applying different methods they were able to participate in the focus-groups which gives the possibility for in depth data collection. We reached consensus through analysing the data according to consensus agreement scores used also by other Delphi studies, i.e. consensus was defined when a large majority agrees on the (very) importance of a health domain. For the health domains, the consensus procedure is described in the part about the Data analysis (consensus was defined as ≥75% of the participants assessed a health domain as important or very important). For the reformulated questions also group consensus was established at 75%, indicating that ≥75 % of the participants evaluated a restated question as better than the original question. | In order to clarify how the focus groups were used within the Delphi rounds, we revised lines 128-135 & 164-167.  ***Within the Delphi procedure, two methods were applied to obtain data within the rounds, namely, an online questionnaire and focus group sessions. These methods were chosen to allow a mixed composition of the panel and the ability to support respondents in their understanding of the questions. Participants with a culturally diverse background or a low level of education were questioned in focus groups during Delphi Rounds 1 and 2. With the support from the experienced focus group facilitators attention for each participant was guaranteed, thus leading to a better understanding of the TOPICS-MDS when discussed with these specific groups instead of using written documents.***  ***The content of the Delphi questionnaire was similar in both the online questionnaire as in the focus groups, although in the focus groups, the 5-point Likert scale was expressed in yellow paper stars that the participants could distribute to assess the importance.*** |
| **- Focus groups are an effective strategy for some qualitative work but the nature of some of the group members may prevent the views of others from being heard. This is inconsistent with the Delphi process.** | We agree with the reviewer that the use of focus groups within the context of a Delphi study could have the risk of unequal contribution to the debate. However, we stress the importance of also involving older adults with low literacy due to a migrant background or a low SES, since those people are often not able to answer online or written questionnaires. If we had used questionnaires, this would probably have led to missing or low quality data within these groups and probably less people being heard.  To ensure the quality of the focus groups and the participation of all participants, we worked with a structured protocol, an experienced focus group facilitator and the presence of trusted key figures during the sessions. Those key figures were themselves part of the target group. They had the trust within their own community and had an affinity with health care. They acted as a bridge between the target group and the researchers.  Some other Delphi studies also describe the use of focus groups as part of a modified Delphi design, for instance in the work of Jeffs et al. (2017) and Ogbeifun & Mbohwa (2016). | Since the pro’s and contra’s of the use of focus groups within a Delphi study is important to discuss, we already addressed this in the Strengths and Limitations section of the original article (line 432-435).  ***However, the use of focus group sessions might have introduced slight bias since the opinions and ratings of others could have influenced the choices of participants, although the advantage of the sessions was valued higher and deep insights were gained.*** |
| **- How is the inherent bias addressed if participants with culturally diverse backgrounds and low education were solely in focus group lines 134-135? This influences the credibility of the quantitative results.** | **See also our reflection above.**  The participants of the focus groups participated fully in round 1 and in round 2 of the Delphi. The third round was solely through an internet questionnaire. This is indeed a limitation of the study we did not previously address since participants with a culturally diverse background or a low level of education were not involved in the third round because this method was considered as not suitable for this response group. | We added this point to the Strengths and Limitations section as a limitation of the quantitative results (435-437).  ***In addition, we did not involve participants with a culturally diverse background or a low level of education in Round 3 of the Delphi study because the method used was a internet questionnaire, which might have influenced the credibility of the quantitative results.*** |
| **- How was the decision made regarding culturally diverse backgrounds and low education (lines 116-118; 134-135)? Define middle level participants please.** | Participants with a culturally diverse background or a low education were approached with the assistance of Pharos (Dutch centre of Expertise on Health Disparities), who have an extensive network and knowledge in these groups.  In the Netherlands there are > 200 different nationalities, and it was therefore impossible to involve all. Furthermore, it is difficult to reach people with a low education to participate in research. Pharos has a broad network of so called 'key figures' in those populations who they approached to contact eligible participants for the focus groups. This guided the final composition of the groups.  Although there a several classification options for levels of education, we chose related to the purpose of this study for a dichotomous classification with a low and a high level of education, which gives an indication if participants would be able to complete a written questionnaire or not. | We supplemented the text in the manuscript to explain about the recruitment of people with culturally diverse backgrounds and low education (line 104 – 111).  ***In the Netherlands, there are over 200 different nationalities; therefore, it was impossible to involve all nationalities. Furthermore, it is difficult to recruit people with a low level of education to participate in research. Pharos has a broad network of 'key figures' in the populations that they approached to find eligible participants for the focus groups. This approach guided the final composition of the groups. The participants with a Hindu background originated mostly from the former Dutch colony of Suriname, and the participants with a Moroccan background were immigrant workers; thus, two different types of migrants in the Netherlands were represented. Highly educated (secondary education, college or university) participants were recruited by a website for older adults (Beteroud.nl) and social media (Facebook and Twitter).***  Further, we clarified the education level in the manuscript as follows: ***Higher educated (secondary education, college or university)*** line 111/112***, Lower education: primary school or less,*** line 233***.*** |
| **- The age of 50 appears quite a young old age and implies that all adults >50 have the same needs.** | Indeed the threshold of ' ≥ 50 years' is not common in research among older adults. However, in this study we chose the threshold of ' ≥ 50 years' deliberately, since research shows that persons with a low education or with a culturally diverse background suffer from multiple chronic conditions at a relative young age (Kunst, 2010) and we wanted to include those groups. In the final sample the mean age range was much higher: 71.5 years. In line with our expectations the mean age for persons with a low education or with a culturally diverse background was lower (resp. 66.7 and 65.8 years) | In the Methods section, sub-heading Participants of the original article, we explained our argumentation for this threshold, related to the characteristics of these populations and our study aim (line 100-102).  ***We deliberately selected the age threshold of ' ≥ 50 years' since research shows that persons with a low level of education or a culturally diverse background suffer from MCC at a relatively young age (******Kunst, 2010******).*** |
| **- Line 235, what does "online panel" mean in table 1 and used in other lines eg. line 134, line 420?** | The online panel refers to the participants that completed the online questionnaire. | We explained this in the relevant sentences and in table 1. |
| **- If the participants identified additional topics (lines 59-60) then is the topics tool actually useful? How might the authors respond or acknowledge this point?** | Since the Dutch Geriatric Society selected the TOPICS-MDS as preferred PROM for older persons with multiple chronic conditions, there is a growing support and use of the TOPICS-MDS in geriatric care in the Netherlands. We think therefore the TOPICS-MDS is useful in our context, especially since the current topics have been assessed as important by ≥ 75% of the older adults in our study. However, the older adults identified some additional topics. | We therefore describe in the Practice Implications that we presented the recommendations regarding the additional topics and the suggestions to improve the comprehensibility of the questions to the Dutch TOPICS Working Group (line 447-448). This working group is continuously working on optimising the TOPICS-MDS. Furthermore, there is currently an implementation programme in the geriatric departments in the Netherlands.  *Practice Implications*  ***The recommendations regarding the additional topics and the suggestions to improve the comprehensibility of the questions have been presented to the Dutch TOPICS Working Group. For the TOPICS-MDS to be used as input for individual healthcare conversations between older adults and healthcare professionals, the education of healthcare professionals is needed. Therefore, in the large scale SDM implementation program of the Dutch Care Institute (Zorg Instituut Nederland), a specific project has been designed to improve SDM with the PROM TOPICS-SF (Short Form), supported by the Dutch Geriatric Society. As a part of this project health professionals at geriatric hospital wards are supported in the use of the results of this PROM in individual health care conversations. For example, through e-learning, they learn how to use the PROM in healthcare conversations. In addition, the Dutch seniors' organisation KBO-PCOB and the Dutch migrant organization NOOM put a joint effort in the empowerment of older adults to prepare for a health care conversation with this PROM, with information sessions for older adults and educational material.*** |
| **- How was the qualitative data analyzed? The authors identify that content analysis was conducted (line 56) but provide no description of the process.** | The qualitative data concerned the in-depth explanation of the answers given in round 1 and round 2 and included open text fragments and audio recordings. The transcripts were analyzed thematically by ‘open coding’ via the program ‘MAXQDA’. Followed by axial coding, where the main themes were clustered and combined. | The analysis of the qualitative data was described in the second part of the paragraph of de Data analysis of the original article (line 213-220).  ***The qualitative data comprised the in-depth explanations of the answers provided in Round 1 and Round 2 and included open text fragments (online questionnaire) and audio recordings (focus groups). The recordings of the focus groups were transcribed verbatim. First, the transcripts were read multiple times, so the reader was familiarized with the data. Subsequently, the transcripts were analyzed thematically by ‘open coding’ via the program ‘MAXQDA’. Following axial coding, the main themes were clustered and combined. Two researchers (RPL and LY) coded the first two transcripts independently and discussed them to reach a consensus. The remaining transcripts were coded by LY and subsequently discussed with RPL.*** |
| **- The term "patient" is often used e.g. lines 365, 370, however, the study was not done with patients** | We changed the term patient to older adult at these lines, as this is attentively noticed by the reviewer. | Term ‘patients’ was changed in ‘older adults’ in the relevant sentences. |
| **Journal Requirements:** | |  |
| Please ensure that your manuscript meets PLOS ONE's style requirements, including those for file naming. | | The manuscript has been checked and revised on PLOS ONE's style requirements. |
| Please include captions for your Supporting Information files at the end of your manuscript, and update any in-text citations to match accordingly. | | The captions for Supporting Information files have been included at the end of the manuscript |
| We would be grateful if you could update the Ethics Statement and Methods section of your manuscript to indicate that the requirement to obtain approval was waived by your ethics committee. | | We changed the Ethics Statement in the Methods section as required (line 141-142):  ***The requirement to obtain approval for this study was waived by the Institutional Review Board of the UMCU (19/355).*** |

Jeffs, L., Kuluski, K., Law, M., Saragosa, M., Espin, S., Ferris, E., . . . Bell, C. M. (2017). Identifying Effective Nurse-Led Care Transition Interventions for Older Adults With Complex Needs Using a Structured Expert Panel. *Worldviews Evid Based Nurs, 14*(2), 136-144. doi: 10.1111/wvn.12196

Kunst, A. E. (2010). *Een overzicht van sociaal-economische verschillen in gezondheid in Europa.* (Vol. 85). Amsterdam: Amsterdam University Press.

Lutomski, J. E., Baars, M. A., Schalk, B. W., Boter, H., Buurman, B. M., den Elzen, W. P., . . . Melis, R. J. (2013). The development of the Older Persons and Informal Caregivers Survey Minimum DataSet (TOPICS-MDS): a large-scale data sharing initiative. *PLoS One, 8*(12), e81673. doi: 10.1371/journal.pone.0081673

Ogbeifun, E., & Mbohwa, C. P., J.H.C. (2016). *Complementing a Delphi exercise with a focus group session*. Paper presented at the Industrial Engineering and Engineering Management, IEEE International Conference on.
